# *N*-Alkane Assimilation by *Pseudomonas aeruginosa* and Its Interactions with Virulence and Antibiotic Resistance

**DOI:** 10.3390/antibiotics13111028

**Published:** 2024-10-31

**Authors:** Balázs Libisch

**Affiliations:** Institute of Genetics and Biotechnology, Hungarian University of Agriculture and Life Sciences, H-2100 Gödöllő, Hungary; libisch.balazs.karoly@uni-mate.hu

**Keywords:** *P. aeruginosa*, alkane hydroxylase, AlkB, hydrocarbon pollution, solvent tolerance, antibiotic resistance, MexAB-OprM

## Abstract

*Pseudomonas aeruginosa* strains with potential for degrading *n*-alkanes are frequently cultured from hydrocarbon-contaminated sites. The initial hydroxylation step of long-chain *n*-alkanes is mediated by the chromosomally encoded AlkB1 and AlkB2 alkane hydroxylases. The acquisition of an additional *P. putida* GPo1-like alkane hydroxylase gene cluster can extend the substrate range assimilated by *P. aeruginosa* to <C12 *n*-alkanes. Efficient niche colonization of hydrocarbon-contaminated sites is facilitated by avid iron-uptake systems, such as pyoverdine, and the production of several compounds with antimicrobial activities. A GPo1-like gene cluster can facilitate detoxification and solvent tolerance in *P. aeruginosa*. The overproduction of various multidrug efflux pumps, in particular, the MexAB-OprM system, can also contribute to solvent tolerance, which is often associated with reduced susceptibility or full resistance to certain clinically relevant antibiotics. These characteristics, together with the remarkable conservation of *P. aeruginosa* virulence determinants among human, animal, and environmental isolates, necessitate further studies from a One Health perspective into the acquired antibiotic resistance mechanisms of environmental *P. aeruginosa* strains and possible ways for their dissemination into the human population.

## 1. Introduction

**Figure 1:**
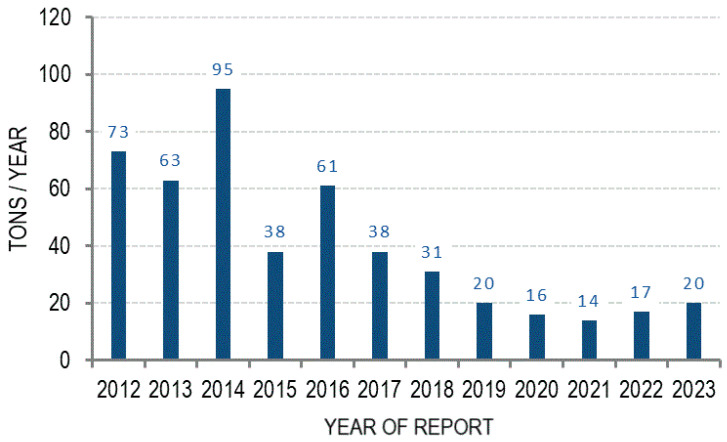
Total petroleum hydrocarbon (TPH) effluents reported annually by MOL Hungarian Oil and Gas Plc. between 2012 and 2023 [2].

Mineral oil (and its constituents, like *n*-alkanes and aromatic hydrocarbons) are among the most important environmental pollutants worldwide as a result of industrial activities and leakage. It is estimated that the worldwide annual spillage of petroleum hydrocarbons exceeds 1.2 million tons [1]. According to the Integrated Annual Reports of the MOL Hungarian Oil and Gas Plc., its total petroleum hydrocarbon (TPH) effluents were in the range of 14 to 95 tons per year between 2012 and 2023, with an overall decreasing trend in most of this period [2]. Efforts by the MOL to improve the quality of discharged waters included, for example, a water recycling project at the Hungarian refinery, and improved sewage systems at Hungarian logistics sites and at the Rijeka Refinery in Croatia [2] (Figure 1). The total volume of oil lost to the environment from tanker spills in 2023 was approximately 2000 tons according to the International Tanker Owners Pollution Federation (ITOPF, https://www.itopf.org/ accessed on 14 October 2024).

Large-scale industrial oil accidents may also contribute to environmental hydrocarbon pollution, such as the accident that took place near Trecate, a small town in the Piedmont region of North-West Italy in February 1994, when a major oil blow-out occurred as a consequence of an accident at an exploration drill-hole. The contamination affected an agricultural area where after intense remediation activity, some zones still remained affected [3]. Taking an example outside of Europe, the Department of Petroleum Resources in Nigeria reported a total of 16,476 oil spills in Nigeria between 1976 and 2015 at different locations in the country, releasing over 3 million barrels of crude oil into the environment [4].

Similar to cyclic hydrocarbons, aliphatic hydrocarbon pollution may be toxic to both soil microorganisms and humans. Shorter alkanes (<C10) partition into the hydrophobic part of the lipid bilayer and thus lower and broaden the membrane transition temperature. The interaction of *n*-alkanes with biological membranes alters their structure and function, both as selective barriers and as a matrix for enzymes. The toxicity of *n*-alkanes is mainly exerted at the level of lipid–lipid and lipid–protein interactions [5,6]. In humans, high concentrations of inhaled alkanes can result in anesthetic effects or narcosis. Among *n*-alkanes, hexane can be considered as the most toxic, where prolonged exposure of humans to *n*-hexane is one of the well-known causes of peripheral neuropathy [7]. A relationship was also proposed between *n*-hexane exposure and the development of defects in color vision [8], while other studies indicated that *n*-hexane and its metabolites may play a role in inducing parkinsonism and hepatotoxic effects [9].

*N*-alkanes with a C12-C18 chain length or longer are degraded by a variety of bacteria; however, the assimilation of shorter-chain alkanes (<C10), which are toxic for the environment, is less frequent [10]. A number of studies reported in the past few decades the frequent isolation of *Pseudomonas* spp. strains from hydrocarbon-contaminated soils, particularly in their first phase of biodegradation, although in certain cases, the Gram-positive bacteria *Rhodococcus* spp. were most abundant [11,12,13]. Among these bacteria, *P. aeruginosa* is one of the most frequently isolated species from hydrocarbon-impacted environments [14,15]. *P. aeruginosa* is a metabolically highly versatile species, allowing it to inhabit a wide range of ecological niches in addition to soil and aquatic environments. This versatility allows *P. aeruginosa* to be both an opportunistic pathogen, chronically colonizing the respiratory tract of cystic fibrosis patients or causing multidrug-resistant nosocomial infections, and an important environmental bacterium degrading ecological pollutants such as detergents or *n*-alkanes [16,17,18]. It would be necessary to examine in more detail the antibiotic resistance and virulence determinants of environmental *P. aeruginosa* isolates mediating *n*-alkane biodegradation, considering their potential role and applications in the bioremediation of hydrocarbon-polluted sites.

A study investigating the microbial community of 19 oil-contaminated soil samples from six major oilfields in China revealed that the dominant genera were *Arthrobacter*, *Dietzia*, *Pseudomonas*, *Rhodococcus*, and *Marinobacter*, where the genera *Pseudomonas* and *Rhodococcus* showed high relative abundances in a large number of samples [19]. Petroleum pollution can cause a decrease in the relative abundances of a range of soil bacteria, with the concurrent enrichment of hydrocarbon-degrading genera such as *Pseudomonas* spp., thereby altering the composition of the dominant soil microbial community [20]. Despite the associated public health risks, *P. aeruginosa* proved to have valuable potential in the remediation of organic pollutants, in particular, for heavy oil-, diesel-, and/or kerosene-polluted water bodies [21]. The challenges raised by the potential emergence and dissemination of antibiotic-resistant strains during the application of *P. aeruginosa* to contaminated soils and other sites have been recognized, and various potential approaches of how to address this issue have been proposed [21,22].

Opportunistic pathogens cause diseases in patients with a predisposition to illness, and a number of opportunistic bacteria originate from terrestrial ecosystems such as the rhizosphere, the zone around plant roots. Due to its high microbial density and rapidly changing conditions, the rhizosphere forms a unique habitat for opportunistic pathogens like *P. aeruginosa*. *Pseudomonas* species are highly competitive for nutrients and several mechanisms involved in the interaction between plants and their associated bacteria are similar to those for pathogenicity in humans [23,24]. Increasing urbanization and the associated use of extensive water distribution systems also contributed to the emergence of *P. aeruginosa* infections, as *P. aeruginosa* can infiltrate urban and hospital water distribution systems and its ability to form biofilms in them may generate a continuous source of contamination [25,26]. This review is aimed at providing a brief overview of the diverse forms of interactions between the pathogenicity and antibiotic resistance of *P. aeruginosa* with its potential to assimilate *n*-alkanes in its natural habitats and on hydrocarbon-contaminated sites.

## 2. Overview of the Species *P. aeruginosa*

The *Pseudomonas* genus comprises Gram-negative, polarly flagellated, aerobic rod-shaped bacteria that are widely distributed in humid environments such as water and soil, and also include certain species that can be pathogenic for plants, while some other species are opportunistic pathogens of animals or humans [27,28]. An important trait of *Pseudomonas* bacteria is the metabolic versatility concerning the carbon sources that these microbes can utilize [22,29,30]. The type species of the genus, *P. aeruginosa* is a motile, non-spore forming, oxidase-positive, and lactose non-fermenter bacterium that produces water-soluble characteristic pigments, the yellow-green and fluorescent pyoverdine and the blue-green pyocyanin. *P. aeruginosa* is also known to cause a variety of hospital infections, including bloodstream infections, pneumonia, surgical-site, and urinary tract infections [31].

*P. aeruginosa* has one of the largest bacterial genomes of about 5.5–7 Mbp [32], which includes a highly conserved core genome (forming about 90% of the whole genome), and the so-called accessory genome. The core genome involves genes that are present in most strains of *P. aeruginosa* (including environmental, clinical, and laboratory strains), and contains a set of metabolic and pathogenic factors [33,34]. About 1/3 of the predicted genes have metabolic functions in the core genome, while others convey cellular process/signaling and information storage/processing functions [35]. The metabolic versatility of *P. aeruginosa* is essentially mediated by various genes of the core genome that are involved in aerobic respiration, denitrification, and anaerobic fermentation. *P. aeruginosa* can grow on a number of different carbon sources, and under anaerobic conditions can also utilize nitrate as a terminal electron acceptor. Proteins associated with other functions, such as regulation, transport, and virulence, may also contribute to the high nutritional versatility of *P. aeruginosa* [36,37].

The accessory genome has been acquired by horizontal gene transfer from different sources, including other bacterial species or genera, and can comprise genes associated, for example, with virulence or antibiotic resistance. Sequences of the accessory genome of *P. aeruginosa* are generally located in extrachromosomal elements, or in blocks of insertions in certain loci [36,38] and are enriched in a variety of mobile genetic elements, including integrative and conjugative elements and phage genes. Likewise, type 1 integrons were also detected in the accessory genome [35].

## 3. Components of the *N*-Alkane Hydroxylase System in *P. aeruginosa*

**Figure 2:**
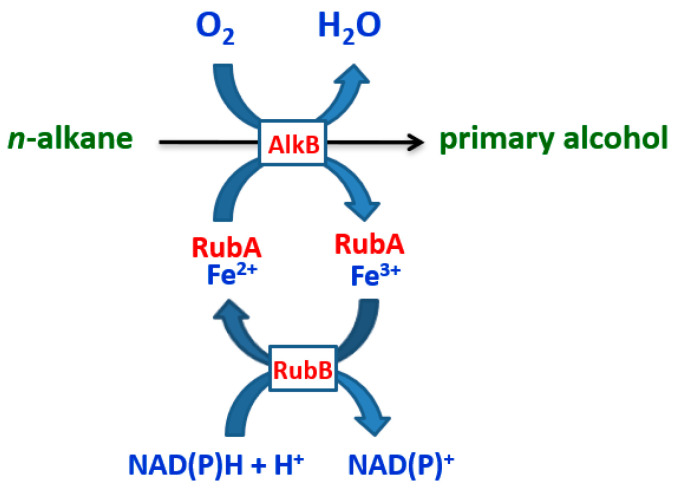
Schematic diagram of the aerobic degradation of *n*-alkanes by *P. aeruginosa*. AlkB, alkane hydroxylase; RubA, rubredoxin; Fe^2+^ and Fe^3+^, the central non-heme iron of RubA; RubB, rubredoxin reductase; NADP, nicotinamide adenine dinucleotide [41,42,43].

*N*-alkanes are chemically inert and thus have to be activated before they can be metabolized. Under aerobic conditions, activation is usually achieved by oxidation of the terminal methyl group to generate the corresponding primary alcohol. This first oxygenation step is catalyzed by the integral membrane enzyme AlkB alkane hydroxylase, containing a non-heme iron center (Figure 2) [39,40] and using one oxygen atom originating from molecular oxygen. The other oxygen atom is reduced to water using two electrons from NAD(P)H [11] (Figure 2). Rubredoxin (RubA) is an essential component of the alkane hydroxylase system. In the *P. aeruginosa* PAO1 reference strain, two rubredoxins (RubA1 and RubA2) are present, which shuttle electrons to the AlkB alkane hydroxylase from a soluble rubredoxin reductase (RubB) at the expense of NAD(P)H (Figure 2) [41,42,43]. Thus, altogether, two alkane hydroxylase (*alkb*1 and *alkB*2), two rubredoxin (*rubA*1 and *rubA*2), and one rubredoxin reductase (*rubB*) genes encode the main functional components of the alkane hydroxylase system of *P. aeruginosa* PAO1. AlkB1 and AlkB2 have overlapping substrate-length profiles: C16 to C24 and C12 to C22 alkanes, respectively. The expression of *alkB2* is highest at the early exponential phase of growth, and when the growth decreases, the *alkB1* gene is induced [41,42,43,44,45].

Most but not all clinical isolates of *P. aeruginosa* assimilate long-chain *n*-alkanes, such as hexadecane, a major component of crude oil, while environmental isolates often grow on medium- as well as long-chain *n*-alkanes [46]. However, clinical *P. aeruginosa* isolates do not grow equally well on long-chain alkanes as environmental strains, because certain factors necessary for alkane degradation may not be optimally expressed in clinical strains [44]. The organization of genes involved in alkane oxidation in *P. aeruginosa* PAO1 is different from that in *P. putida*. The two chromosomal alkane hydroxylase genes *alkB1* and *alkB2* in the *P. aeruginosa* PAO1 genome are not in close proximity of the genes coding for the electron transfer proteins RubA and RubB [47]. For comparison, the *P. putida* strains GPo1 and P1 possess two operons, which contain all genes involved in alkane degradation [48].

## 4. *P. aeruginosa* Isolates with an Ability to Degrade Medium-Chain *N*-Alkanes

As opposed to the substrate range of the *P. aeruginosa* AlkB1 and AlkB2 enzymes (C12–C16), the *P. putida* strain GPo1 AlkB can oxidize medium-chain alkanes containing 5 to 12 carbon atoms [48]. However, the substrate specificity of the latter enzyme is relaxed, and it can oxidize other molecules as well, including propane and butane gases, alcohols, and catalyzes demethylation and sulfoxidation reactions. The propane and butane gases, and not their corresponding primary alcohols, also act as inducers of alkane-oxidizing activity [49]. While *P. putida* GPo1 oxidizes <C12 alkanes, most alkane-degrading bacteria such as strains of *P. aeruginosa* usually assimilate alkanes containing 12 to 20 or sometimes up to >30 carbon atoms [50].

**Figure 3:**
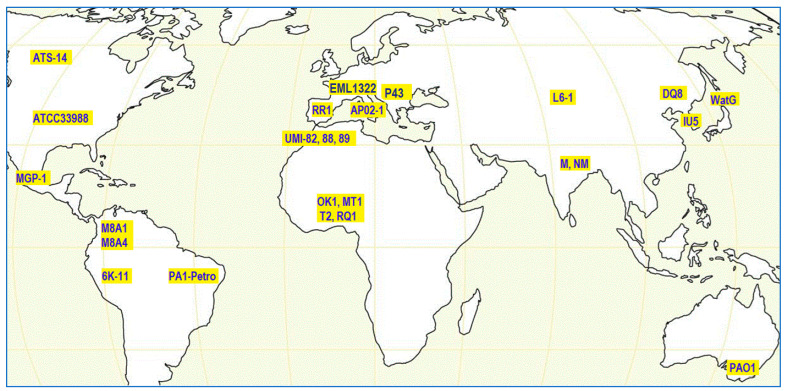
Geographical distribution of selected *P. aeruginosa* strains that were isolated globally from hydrocarbon-impacted environments. *P. aeruginosa* strains and their countries of origin are ATS-14 (Canada), ATCC33988 (USA), MGP-1 (Mexico), M8A1 and M8A4 (Colombia), 6K-11 (Peru), PA1-Petro (Brazil), RR1 (Spain), EML1322 (France), AP02-1 (Italy), P43 (Hungary), UMI-82, UMI-88, and UMI-89 (Morocco), OK1, MT1, T2, and RQ1 (Nigeria), L61 and DQ8 (China), M and NM (India), IU5 (South Korea), WatG (Japan), and the clinical reference strain PAO1 (Australia) [51,52,53,54,55,56,57,58,59,60,61,62,63,64,65,66].

The *alkB1* and *alkB2* alkane hydroxylase genes were present in all environmental and clinical isolates of *P. aeruginosa* strains tested in a comparative study [46]. Figure 3 shows the global distribution of selected *P. aeruginosa* strains isolated from hydrocarbon-impacted environments in various countries, together with the reference clinical PAO1 strain that also assimilates *n*-alkanes [51,52,53,54,55,56,57,58,59,60,61,62,63,64,65,66]. Interestingly, *P. aeruginosa* strains isolated from gasoline spills with C6 or C8 alkanes as sole carbon sources often contain an additional *alk* gene that is (almost) identical to that of the *P. putida* GPo1 *alk* system [67]. These GPo1-type *alk* gene-positive *P. aeruginosa* strains can grow on C5–C11 alkanes in addition to C12–C16 alkanes. The typical composition of gasoline contains mainly alkanes and other carbohydrates with <12 carbon atoms [68,69,70].

In accordance with these findings, only 3 out of 20 *P. aeruginosa* strains isolated in Morocco from an oil-polluted site had the ability to use C6-C10 alkanes. The presence of an *alkB* gene sharing a high level of similarity with that of *P. putida* GPo1 was detected only in these three strains designated UMI-82, UMI-88, and UMI-89 (Figure 3). The acquisition of a GPo1-like gene by *P. aeruginosa* extended the spectrum of their alkane utilization from C12–C22 to shorter chain lengths as well [67].

Alkane-degrading bacteria normally thrive in unpolluted environments where alkanes synthesized by algae, plants, and other organisms are present at low concentrations [50]. For example, plants produce *n*-alkanes as natural constituents of waxes, where their amount varies from trace levels to forming the major constituent of the plant wax [71]. The presence of alkane hydroxylases in the genome sequences of *P. aeruginosa*, and other bacteria such as *L. pneumophila* and *B. pseudomallei,* may therefore reflect the opportunistic pathogen character of these environmental microbes [72]. The chromosomally encoded AlkB1 and AlkB2 hydroxylases may be sufficient to degrade alkanes that *P. aeruginosa* strains normally encounter in their natural environment. The acquisition of a GPo1-like hydroxylase system may be favored and selected by high amounts of medium-chain alkanes, for example, at petroleum-contaminated sites. From this respect, it is interesting that even in some clinical *P. aeruginosa* isolates, an *alkS* gene (a regulator of the GPO1-like gene cluster) was detected, indicating the presence of a GPo1-like hydroxylase system [73]. These strains may have possibly disseminated to the human population from petroleum-contaminated environments.

Alkanes are not preferred carbon sources for *P. aeruginosa*. Alkane oxidation activity in various *P. aeruginosa* strains has been reported to be subject to carbon catabolite repression by glucose and other carbon sources [74]. The induction of alkane oxidation in intact cells of *P. aeruginosa* was repressed by glucose where a maximal repression of 80% was attained at a glucose concentration as low as 0.1%. Induction could be completely repressed by malate [75].

The GPO1-like hydroxylase pathway is induced to very high levels in the presence of alkanes through the AlkS regulator, which is detrimental to cell physiology. However, a fast switch-off of the system in the absence of alkanes saves the metabolically expensive NADH for other purposes [76,77]. The sequence of the *alkS* gene has 44.8% GC content, which is in clear contrast to the overall 67% GC content of the *P. aeruginosa* genome. Likewise, the *alk* genes in *P. putida* have GC contents of between 44 and 47%, as opposed to 61.5% for the *P. putida* genome. These data indicate that a GPO1-like gene cluster was only recently acquired by the *Pseudomonas* genus. Accordingly, out of 11 clinical *P. aeruginosa* strains examined, only 1 possessed this gene and its transcript [73]. *Alcanivorax borkumensis*, an alkane-degrading marine bacterium, features an *alk* gene with high similarity to that of *P. putida* strain GPo1, thus providing support for its proposed recent horizontal gene transfer [41,78].

## 5. Interactions Between Microbial Ecology and *N*-Alkane Assimilation by *P. aeruginosa*

*P. aeruginosa* strains produce rhamnose-containing glycolipid biosurfactants, the so-called rhamnolipids. Two major glycolipid types are produced in liquid cultures, referred to as mono-rhamnolipids and dirhamnolipids, respectively [79]. Rhamnolipids enhance the degradation of hydrocarbons in two different ways. They increase the solubility and bioavailability of hydrocarbons, and also interact with the bacterial cells [80]. It was proposed that an alternative function of rhamnolipids is distinct from facilitating the assimilation of insoluble substrates, as these molecules are also efficiently produced when the *P. aeruginosa* cells are grown on soluble substrates. An ecological role of rhamnolipids may be explained by their toxicity against a variety of bacteria, mostly against Gram-positives and also against some Gram-negatives, which confers a competitive advantage in niche colonization. Other antimicrobial activities including antifungal, algicidal, and antiamoebal have also been reported [81,82].

The biodegradation of hydrocarbons generally involves iron-containing oxygenase enzymes. Therefore, iron-limited environments may influence the rate of their degradation [83]. *P. aeruginosa* strains possess multiple iron acquisition systems to thrive in low iron niches. Iron-dependent transcriptional responses were reported in known iron acquisition systems in *P. aeruginosa* including pyoverdine, pyochelin, and heme uptake. Pyoverdine has high affinity for Fe^3+^ ions and is a siderophore (iron carrier) of the producer strain. In iron-depleted media in vitro, the pyoverdine-producing *Pseudomonas* spp. strains inhibit the growth of other bacteria and fungi with less potent siderophores. Therefore, under certain conditions, pyoverdine may function as a diffusible bacteriostatic or fungistatic antibiotic [84]. The potent iron acquisition system of *P. aeruginosa* may provide a selective advantage in hydrocarbon-contaminated environments for the synthesis of the iron-containing alkane degradative enzymes and can provide a tool to compete with other microorganisms that have less efficient iron-uptake systems [85].

## 6. Interactions Between *N*-Alkane Assimilation and Antibiotic Resistance

Antibiotic resistance on hydrocarbon-contaminated sites may emerge as a result of the acquisition of *n*-alkane tolerance. The MexAB-OprM, MexCD-OprJ, and MexEF-OprN multidrug efflux pumps of *P. aeruginosa* were reported to also accommodate various organic solvents [86,87]. In the presence of *n*-hexane, organic solvent-tolerant mutants of *P. aeruginosa* were selected, which had increased minimal inhibitory concentrations against β-lactams, fluoroquinolones, chloramphenicol, tetracycline, and novobiocin. The solvent-tolerant mutants showed elevated versus decreased expression of the *mexAB-oprM* and *mexEF-oprN* systems, respectively. Mutations in *mexR*, the repressor gene of the *mexAB-oprM* efflux operon, were identified in two solvent-tolerant mutant strains, indicating the importance of the MexAB-OprM efflux system in solvent tolerance (see also Table 1, Ref. [86]). *N*-alkanes, as environmental pollutants, may thus contribute to the selection of *P. aeruginosa* strains with an antibiotic-resistant phenotype [86,87].

In accordance with these results, in another study, the MexAB-OprM system was found to be far the superior efflux system providing solvent tolerance in *P. aeruginosa* [88]. Solvent tolerance was compromised by a protonophore, suggesting that it is an energy-dependent mechanism. As *n*-alkanes dissolve in lipid membranes and their toxic effect involves perturbations of the cytoplasmic membrane function, the efflux systems may potentially access organic solvents from within the cell membrane bilayer as well [89]. MexAB-OprM from *P. aeruginosa* can remove *n*-hexane and *p*-xylene as well as antibiotics, including β-lactams and tetracycline. Deletion of the *mexA-mexB-oprM* operon or of the *oprM* gene alone rendered the examined *P. aeruginosa* strain unable to grow in the presence of these solvents. The MexAB-OprM, MexCD-OprJ, and MexEF-OprN pumps were shown to also contribute to multidrug resistance in *P. aeruginosa*, as they can accommodate structurally diverse substrates [89,90]. Genes related to alkane degradation, membrane proteins, and efflux pumps were upregulated during growth in jet fuel in *P. aeruginosa* strain ATCC 33988 isolated from a fuel tank in Oklahoma, suggesting that transcriptional control is a main mechanism for the adaptation of *P. aeruginosa* to hydrocarbon-contaminated environments [91].

Growth on hexadecane as a sole carbon source did not significantly alter or increase the antibiotic resistance of twelve *P. aeruginosa* isolates of various origin, including two clinical isolates [92]. These observations suggest that growth on medium-chain *n*-alkanes, such as hexane, may primarily contribute to the selection of efflux-mediated antibiotic resistance in *P. aeruginosa*, as opposed to growth on long-chain alkanes (see examples in Table 1).

**Table 1:** Examples for *P. aeruginosa* strains displaying increased MICs against various antibiotics in response to hydrocarbon exposure ^a^.

Strain	Increased MICs Against Antibiotics Including ^b^	Reported Resistance Mechanisms	References
K1261	CAR, FEP, CIP, TET, CHL	Increased expression of *mexAB–oprM*, mutation in *mexR*	[86,88]
K1262	CAR, FEP, CIP, TET, CHL	Increased expression of *mexAB–oprM*, mutation in *mexR*	[86,88]
Po10	AMP, KAN	Antibiotics pumped out by multidrug efflux systems	[93]
Po14	AMP, KAN	Antibiotics pumped out by multidrug efflux systems	[93]
RR1	CIP, NOR, CAZ, ERY, CHL	Expression of multidrug resistance efflux pump systems	[46]
CECT119	CIP, NOR, STR, ERY, CHL	Expression of multidrug resistance efflux pump systems	[46]
ATCC21472	CAZ, ERY, TET, CHL	Expression of multidrug resistance efflux pump systems	[46]
K1542	NOR, ERY	Increased expression of *mexCD-oprJ* induced by *n*-hexane	[94]

^a^ Abbreviations of antibiotics: AMP, ampicillin; CAR, carbenicillin; CAZ, ceftazidime; CHL, chloramphenicol; CIP, ciprofloxacin; ERY, erythromycin; FEP, cefepime; KAN, kanamycin; NOR, norfloxacin; STR, streptomycin; TET, tetracycline. ^b^ MIC stands for minimal inhibitory concentration.

Four *P. aeruginosa* isolates were isolated from crude oil-contaminated effluent in the Niger delta area of Nigeria, which displayed resistance to amikacin, gentamicin, carbenicillin, and/or chloramphenicol. The multiple antibiotic resistance of these isolates had a relevance from the clinical standpoint as well, to be tested and considered before the use of such strains in biocontrol or bioremediation processes. *P. aeruginosa* strain T2 had the most notable antibiotic resistance profile with no inhibitory zones for amikacin and gentamicin in a disk diffusion assay, indicating a high level of resistance against these important drugs (Table 2) [95]. Multidrug-resistant *P. aeruginosa* strains were also isolated in Hungary from hydrocarbon-impacted environmental samples, with a high level of MICs for several of the commonly used anti-pseudomonas antibiotics (Table 2) [65].

*P. aeruginosa* strains treated with sub-inhibitory concentrations of netilmicin displayed decreased cell surface hydrophobicity and decreased attachment to *n*-hexadecane compared to non-treated isolates. Inhibition of hydrophobicity by aminoglycosides, such as by netilmicin or gentamicin, was proposed to be a result of strain susceptibility and of the pleotropic effect of ribosomal function and membrane damage [96]. A similar effect was observed by treatment with a sub-inhibitory concentration of β-lactam antibiotics that also induce changes in the surface properties of *P. aeruginosa* isolates [97]. Therefore, resistance to various antibiotics may be a selective advantage for *P. aeruginosa* isolates to maintain sufficient levels of cell surface hydrophobicity and attachment to hydrocarbon substrates on contaminated sites.

**Table 2:** Examples for antibiotic-resistant *P. aeruginosa* strains isolated from hydrocarbon-impacted environmental sites of various geographical locations ^a^.

Country	Location	Sample Type	Strain	Reported Resistance ^b^	Ref.
France	Neuves Maisons	HC-impacted soil ^c^	EML1321	TIC, TIM	[66]
France	Neuves Maisons	HC-impacted soil	EML1322	TIC, TIM, IPM	[66]
Hungary	Ópusztaszer	HC-polluted groundwater	P43	PIP, CAZ, FEP, IPM, GEN	[65]
Hungary	Nagyszénás	HC-polluted groundwater	P69	PIP, CAZ, IPM	[65]
Nigeria	South Nigeria	Crude oil-polluted site	01	CAZ	[98]
Nigeria	Niger delta	Crude oil-polluted effluent	T2	GEN, AMK	[95]
Nigeria	Ado	Diesel-polluted soil	SGHB7	CAZ	[99]
Nigeria	Lagos	Diesel generator site soil	LP6	GEN	[100]

^a^ Resistant phenotypes are shown here only for clinically relevant anti-pseudomonas agents of the reported antibiograms, with the abbreviations as follows: AMK, amikacin; CAZ, ceftazidime; FEP, cefepime; GEN, gentamicin; IPM, imipenem; PIP, piperacillin; TIC, ticarcillin; TIM, ticarcillin/clavulanic acid. ^b^ See text for further details on gentamicin. ^c^ HC stands for hydrocarbon.

Antibiotics pollution in aquatic and terrestrial environments can provide an additional selection pressure for the emergence of antibiotic-resistant *P. aeruginosa* strains [101]. The emergence of acquired antibiotic resistance mechanisms in *P. aeruginosa* through genetic mutations and/or by horizontal gene transfer on crude oil-contaminated sites may be further promoted by the highly mutagenic polycyclic aromatic hydrocarbons [102,103]. Selected *P. aeruginosa* strains isolated from hydrocarbon-contaminated environmental samples are shown in Table 2, displaying resistant phenotypes against various clinically relevant anti-pseudomonas agents that were tested in vitro in their referenced studies. CLSI and EUCAST do not currently publish gentamicin breakpoints for *P. aeruginosa* [104,105]; however, gentamicin is the subject of several studies to enhance its antimicrobial efficacy and drug delivery in potential clinical applications against *P. aeruginosa* infections [106,107,108,109].

## 7. Relationships Between Pathogenicity and *N*-Alkane Assimilation

### 7.1. Virulence Determinants of Human, Animal, and Environmental P. aeruginosa Isolates

Infections caused by *P. aeruginosa* in the airways of patients suffering from cystic fibrosis (CF) usually derive from the environment [110]. A study of 12 hydrocarbon-degrading *P. aeruginosa* strains of environmental (soil, water) and human origin revealed that all tested strains expressed virulence factors [92]. Likewise, the presence of virulence determinants among human, bovine, and groundwater isolates of *P. aeruginosa* was compared by PCR screening for genes *toxA* (exotoxin A), for the type III secretion system (T3SS) effector genes *exoS*, *exoT*, *exoY*, and *exoU*, and for *algD* (GDP-mannose dehydrogenase involved in alginate biosynthesis and biofilm formation) [111]. The distribution of these virulence determinants was very similar: all genes were present in the three major sources, except for *exoU*, which was only detected in one human and one groundwater isolate. The lung invasion potential in mouse lung inoculation experiments 20 h after intranasal application was 66%, 78%, and 53% for isolates with human, bovine, and groundwater origin, respectively [111]. *P. aeruginosa* environmental isolates examined in another study (including strains from hydrocarbon-impacted environments) produced proteases, were able to invade epithelial cells in a cell culture model system, and also carried genes from a type III secretion system [46]. Given that environmental *P. aeruginosa* isolates showed several virulence properties similar to those of the clinical isolate PAO1 [46,92,111], it is necessary to explore potential relationships between their pathogenicity and *n*-alkane assimilation from a One Health perspective.

### 7.2. The Impact of Rhamnolipids and Cell Surface Hydrophobicity on Hydrocarbon Assimilation and Virulence

Rhamnolipids contain a sugar moiety (a monomeric or dimeric rhamnose unit) linked to β-hydroxylated fatty acid chains and are biosurfactants because of their amphiphilic nature [112]. Rhamnolipid synthesis is regulated by the quorum-sensing system in *P. aeruginosa*, a mechanism that controls the production of many virulence factors [113]. Rhamnolipids cause membrane damage in human lung epithelial cells and exhibit highly acute toxicity towards murine macrophages, where macrophage death was strongly correlated to rhamnolipid production [114]. Thereby, rhamnolipids can facilitate evading the host-induced phagocytosis of *P. aeruginosa* cells [115]. Rhamnolipid production by colonizing *P. aeruginosa* isolates was linked to the development of ventilator-associated pneumonia in mechanically ventilated patients [116].

The *P. aeruginosa* lipopolysaccharide (LPS) molecules of the outer membrane influence the cell surface properties, where the O-antigen component of LPS is in contact with the environment and impacts cell surface hydrophobicity [14]. It was proposed that the O-antigen has a regulating role in the *P. aeruginosa* aggregates’ size and shape in cystic fibrosis airways by altering the relative hydrophobicity of the cell surface [117]. *P. aeruginosa* may adapt to cystic fibrosis airways by the loss of the O-antigen, leading to changes in cell surface hydrophobicity, where cells lacking the O-antigen can assemble into clumped aggregates [118]. The airway mucus in cystic fibrosis has a higher (*p* < 0.05) lipid content than normal mucus, and this higher lipid content seemed to be related to the degree of infection [119,120,121].

In hydrocarbon-impacted environmental niches, rhamnolipids contribute to the uptake and assimilation by *P. aeruginosa* of the hydrophobic *n*-alkanes, such as hexadecane, through increasing the cell surface hydrophobicity and thereby increasing adhesion to hydrocarbons and bacterial cell-to-cell aggregation by hydrophobic interactions [122]. Rhamnolipids caused higher cell surface hydrophobicity by releasing lipopolysaccharide (LPS) from the outer membrane of *Pseudomonas* bacteria [81]. The amendment of rhamnolipids produced by *P. aeruginosa* to oil sludge containing soil enhanced the biodegradation of *n*-alkanes in microcosm experiments [123]. Rhamnolipids were also found to be necessary for biofilm formation and maintenance, and for establishing and sustaining fluid channels in biofilms for the transport of water and oxygen [122].

The petroleum-degrading *P. aeruginosa* strain WatG was isolated in Hokkaido, Japan, from water in an old kerosene tank (Figure 3) [124]. In soil microcosm experiments containing strain WatG, the production of dirhamnolipids was observed only in the presence of diesel oil, indicating that diesel oil served as an inducer of dirhamnolipid synthesis and secretion into the soil [125]. The *rhlAB* operon encoding a rhamnosyltransferase enzyme involved in rhamnolipid synthesis was upregulated over 5-fold in response to Jet A fuel in *P. aeruginosa* strain ATCC 33988, originally isolated from a fuel storage tank [126] (Figure 3). Further, *P. aeruginosa* isolates grown on Bonny Light crude oil showed reduced O-antigen expression and an associated increased cell surface hydrophobicity [14].

Taken together, these observations point to a role of elevated cell surface hydrophobicity both in hydrocarbon assimilation and in certain human infections caused by *P. aeruginosa* and suggest that high levels of hydrocarbon-induced rhamnolipid production may be potentially associated with a higher level of virulence of such *P. aeruginosa* isolates.

### 7.3. The Diverse Effects of Efflux Pumps and Their Overproduction

It was reported that mutations in *mexR* (and potentially also in *nalD* or *nalC*) can emerge both under antibiotic and *n*-alkane selection, leading to the overproduction of the MexAB-OprM efflux pump, and an associated decreased susceptibility or resistance of *P. aeruginosa* against various antibiotics [86,88,127] (Table 1). Overproduction of MexAB-OprM increases the MICs (besides other agents) of most β-lactam antibiotics, including meropenem, and its overexpression is globally frequent among *P. aeruginosa* clinical isolates [128].

Certain *P. aeruginosa* strains are able to transmigrate through epithelial cells and cause invasive human or animal diseases [129]. Determinants of invasion were proposed to be exported from the cell by the MexAB-OprM efflux system [130]. A possible reason for the attenuated virulence associated with the overexpression of efflux pumps is that these pumps also extrude quorum-sensing signals from the *P. aeruginosa* cells, and such lowering of their intracellular concentration hampers the efficient production of different virulence factors [131]. Therefore, it was suggested that normal efflux levels are optimal for *P. aeruginosa* for its maximal invasiveness [129].

On the other hand, *P. aeruginosa* mutants overexpressing *mexAB-oprM* and *mexCD-oprJ* did not display defects in biofilm development. Instead, *nalB* mutant strains showed significantly denser biofilm formation compared to wild-type, where the overexpression of *mexAB-oprM* was proposed to enhance the efflux of acyl-homoserine lactones (signaling molecules involved in quorum sensing) and thus mediate rapid biofilm formation [132]. Also, an upregulated efflux may protect *P. aeruginosa* cells by exporting waste metabolites produced within the biofilm during anaerobic respiration. Accordingly, *P. aeruginosa* biofilm formation can be inhibited by the application of efflux pump inhibitors [133].

### 7.4. The Role of Biofilms in N-Alkane Assimilation and Pathogenesis

In its diverse environmental habitats, *P. aeruginosa* can use different survival strategies, like developing biofilms on high-temperature oilfields [134]. When a suitable attachment site is available, *P. aeruginosa* switches from a planktonic state to biofilm development. Planktonic cells are immobilized, and cell to cell communication by the quorum-sensing system results in their aggregation and formation of a microbial biofilm community [115].

Lastly, exopolysaccharides are produced to develop and stabilize the three-dimensional biofilms [135]. *P. aeruginosa* can produce during biofilm formation, in addition to alginate, two further exopolysaccharides: PSL and PEL [136].

It was shown that biofilm-amended cultures of *Pseudomonas* isolates could assimilate individual hydrocarbons by a 20–40% greater efficiency in comparison to planktonic cells alone [137]. When fuel tank or pipeline surfaces also contain water, inorganic particles, and sufficient nutrients, biofilm development can be initiated. Biofilms are usually attached to the inner surfaces of fuel storage facilities, fuel/water interfaces, or to sediments at the bottom of fuel tanks. *P. aeruginosa* and other *Pseudomonas* species have been isolated in the USA from commercial and military aviation fuel facilities since 1958 [138,139].

Similar to biofilms that may be formed by different *P. aeruginosa* strains in hydrocarbon-impacted environments, in the respiratory tracts of cystic fibrosis patients, *P. aeruginosa* can also overproduce extracellular polysaccharide materials such as alginate and develop biofilms [115,126,140]. *P. aeruginosa* biofilms can be associated with reduced cough clearance from the lung, and for these reasons, viscoelasticity was suggested to be considered as a virulence trait of such biofilms [141]. The formation of *P. aeruginosa* biofilms can induce chronic and recurrent human infections, in particular, wound infections, lung infections, or catheter infections.

Overall, the biofilm-forming potential of both environmental and clinical *P. aeruginosa* isolates should be considered as an important factor contributing to their efficient hydrocarbon assimilation and/or pathogenicity in certain human infections. An increased expression of the *mexAB-oprM* system due to mutations in *mexR* or other regulators might also contribute to the potent biofilm formation of *P. aeruginosa*.

### 7.5. Other Related Characteristics of P. aeruginosa Isolates

The competition between bacterial cells for iron uptake in alkane-enriched environments is in many aspects similar to *P. aeruginosa* infections, when the bacterium produces siderophores, such as pyoverdine, to obtain iron, under strong competition with the human host. Human pathogenic bacteria need to be able to acquire iron from host tissues, where it is tightly bound to transferrin or, in the airways, lactoferrin. Pyoverdine and other siderophores are therefore considered to be essential for bacterial virulence. Indeed, siderophore-deficient mutants of pathogenic bacteria are less virulent in disease models [142,143,144,145,146].

It is important to highlight that many characteristics of *P. aeruginosa* that were briefly discussed in this review depend upon important strain-specific phenotype and/or genotype variations among characterized *P. aeruginosa* isolates, including the presence of genes encoding additional long-chain alkane hydroxylases [134], the influence of strain-specific genotypes on the impact of *mexAB-oprM* overexpression on virulence [147], the effect of efflux pump inhibitors on *P. aeruginosa* virulence in vivo [148], and several others.

**Figure 4:**
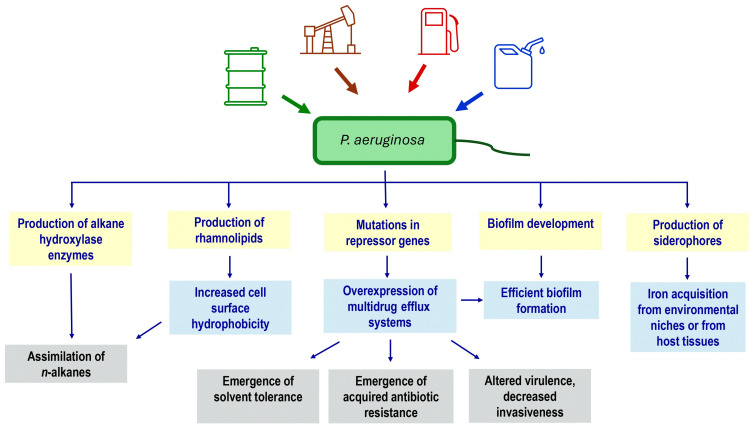
Selected potential interactions between *n*-alkane assimilation by *P. aeruginosa* and its virulence and/or antibiotic resistance. See text for further details and information.

Although environmental and clinical *P. aeruginosa* strains share the vast majority of their genes, coordinate transcriptional control plays a prominent role in niche adaptation. The *P. aeruginosa* genome encodes over 500 transcriptional regulators, making up nearly 10% of its coding capacity, and post-transcriptional regulation can also control protein levels in *P. aeruginosa* [149]. The remarkable conservation of genes encoding virulence factors indicates that most strains, including soil and groundwater isolates of *P. aeruginosa,* may serve as the source for a wide variety of human infections [46,92,111]. A graphical summary for selected potential interactions between the *n-*alkane assimilation by *P. aeruginosa* and its virulence and antibiotic resistance mechanisms is shown in Figure 4.

## 8. Conclusions

*P. aeruginosa* strains are frequently cultured from hydrocarbon-contaminated sites with potential for degrading aliphatic hydrocarbons. Most *P. aeruginosa* isolates, regardless of their source, mineralize C12–C16 alkanes, where the initial hydroxylation step is mediated by the chromosomally encoded non-heme iron-containing AlkB1 and AlkB2 alkane hydroxylases. The acquisition of an additional GPo1-like alkane hydroxylase gene cluster can extend the assimilated substrate range of *P. aeruginosa* to shorter (<C12) *n*-alkanes as well. Efficient niche colonization of hydrocarbon-contaminated sites by *P. aeruginosa* is facilitated by avid iron-uptake systems, such as pyoverdine, and the production of compounds with antimicrobial activities, including pyoverdine, rhamnolipids, and pyocyanin. In addition to fuel assimilation, a key function of these acquired GPo1-like gene clusters is detoxification and contribution to the solvent tolerance of *P. aeruginosa*. Elevated solvent tolerance can also be mediated by the overproduction of multidrug efflux pumps, in particular, the MexAB-OprM system, where their overproduction often results in reduced susceptibility or full resistance to a range of clinically relevant antibiotics. These characteristics, together with the remarkable conservation of *P. aeruginosa* virulence determinants among human, animal, and environmental isolates, point to potential public health risks associated with the application of *P. aeruginosa* for the in situ bioremediation of hydrocarbon-contaminated sites. Further studies are needed to explore the acquired antibiotic resistance repertoire of environmental *P. aeruginosa* isolates and possible ways for their dissemination into the human population from a One Health perspective [150,151].

## Data Availability

No new data were created or analyzed in this study.
